# Serotype Distribution and Antibiotic Susceptibility of *Streptococcus pneumoniae* Strains Carried by Children Infected with Human Immunodeficiency Virus

**DOI:** 10.1371/journal.pone.0110526

**Published:** 2014-10-24

**Authors:** Dodi Safari, Nia Kurniati, Lia Waslia, Miftahuddin Majid Khoeri, Tiara Putri, Debby Bogaert, Krzysztof Trzciński

**Affiliations:** ^1^ Eijkman Institute for Molecular Biology, Jakarta, Indonesia; ^2^ Department of Child Health, Dr. Cipto Mangunkusumo Hospital/Faculty of Medicine Universitas Indonesia, Jakarta, Indonesia; ^3^ Eijkman Oxford Clinical Research Unit, Jakarta, Indonesia; ^4^ Faculty of Biology, Gajah Mada University, Yogyakarta, Indonesia; ^5^ Department of Pediatric Immunology and Infectious Diseases, Wilhelmina?s Children Hospital, University Medical Center Utrecht, Utrecht, the Netherlands; Columbia University, United States of America

## Abstract

**Background:**

We studied the serotype distribution and antibiotic susceptibility of *Streptococcus pneumoniae* isolates carried by children infected with HIV in Jakarta, Indonesia.

**Methods:**

Nasopharyngeal swabs were collected from 90 HIV infected children aged 4 to 144 months. *S. pneumoniae* was identified by conventional and molecular methods. Serotyping was performed with sequential multiplex PCR and antibiotic susceptibility with the disk diffusion method.

**Results:**

We identified *S. pneumoniae* carriage in 41 children (46%). Serotype 19F was most common among 42 cultured strains (19%) followed by 19A and 6A/B (10% each), and 23F (7%). Most isolates were susceptible to chloramphenicol (86%), followed by clindamycin (79%), erythromycin (76%), tetracycline (43%), and sulphamethoxazole/trimethoprim (41%). Resistance to penicillin was most common with only 33% of strains being susceptible. Strains of serotypes targeted by the 13-valent pneumococcal conjugate polysaccharide vaccine (PCV13) were more likely to be multidrug resistant (13 of 25 or 52%) compared to non-PCV13 serotype isolates (3 of 17 or 18%; Fisher exact test *p* = 0.05).

**Conclusion:**

Our study provides insight into the epidemiology of pneumococcal carriage in young HIV patients in Indonesia. These findings may facilitate potential preventive strategies that target invasive pneumococcal disease in Indonesia.

## Introduction

*Streptococcus pneumoniae* is a leading cause of bacterial pneumonia, meningitis, and sepsis worldwide. An estimated 1.6 million people die from invasive pneumococcal disease (IPD) each year, one million of whom are children [1]. Incidence of IPD varies substantially by age, genetic background, socioeconomic status, immune status, and geographical location [2]. Capsular polysaccharide is considered to be the ultimate virulence factor of *S. pneumoniae* as un-encapsulated strains are virtually absent among *S. pneumoniae* causing IPD [3],[4]. Over 90 *S. pneumoniae* serotypes have been identified based on the capsule chemical structure and immunogenicity [5] and capsular oligosaccharides are used as vaccine antigens in pneumococcal vaccines.

Current pneumococcal conjugate vaccines cover only a selected set of serotypes, e.g. PCV7 (7 serotypes), PCV10 (10 serotypes) and PCV13 (13 serotypes). The introduction of the PCV7 vaccine targeting the serotypes 4, 6B, 9V, 14, 18C, 19F, and 23F significantly reduced the burden of pneumococcal disease in many populations [6]. Despite high efficacy against disease caused by the vaccine serotypes (VTs), the net effect of vaccination is often reduced due to serotype replacement [6],[7]. In a number of geographical locations including the USA, Germany, The Netherlands, England and Wales [8]–[11], serotype 19A was reported to be the most commonly emerging non-vaccine serotype (NVT) following PCV7 introduction. Colonization of the upper respiratory tract is the obligatory first step in the pathogenesis of pneumococcal disease, and therefore considered the most important risk factor for IPD [12]. It also provides the basis for horizontal spread of pneumococci in the community, making it an important target for preventive measures [13],[14].

Currently, epidemiological data on *S. pneumoniae* carriage and invasive disease is limited for the Indonesian population. In 2001, Soewignjo *et al*. reported that the prevalence of *S. pneumoniae* carriage was 48% in healthy children in Lombok Island, Indonesia [15]. Recently, Farida *et al.* reported that in Semarang, Indonesia, prevalence of *S. pneumoniae* in 2010 was 43% and 11% in children aged 6–60 months and adults aged 45–75 years, respectively [17]. One of the risk factors for IPD is infection with human immunodefficiency virus (HIV) [16]. So far, no data are available on *S. pneumoniae* carriage in high-risk populations in Indonesia. Currently, pneumococcal vaccination is not part of the expanded program on immunization (EPI) for infants in Indonesia. Both the PCV13 (targeting all PCV7 serotypes plus serotypes 1, 3, 5, 6A, 7F and 19A) and the 23-valent pneumococcal polysaccharide vaccine (PPV23) are available at a commercial price. The use of pneumococcal vaccines is not monitored in any systemic way in Indonesia.

In this present study, we investigate the carriage of *S. pneumoniae* in children infected with HIV in Jakarta, Indonesia. We expect our results to guide the modification of existing, and the implementation of potentially new preventive strategies targeting pneumococcal disease in the country.

## Methods

### Study population

A cross-sectional study on *S. pneumoniae* nasopharyngeal colonization was performed from January to July 2012 among children infected with human immunodeficiency virus (HIV) during their routine clinic visits at the Cipto Mangunkusumo Hospital, Jakarta – Indonesia. The study has been reviewed and approved by the ethical committee of Faculty of Medicine, Universitas Indonesia, Jakarta, Indonesia. The children's parents signed informed consent forms and provided clinical and demographic information, such as age, sex, family size and in which region they were living. Detailed medical information on the CD4 lymphocyte count within the past 3 months and the use of antibiotics was recorded during the study. Parents were also asked whether any person living with a child is smoking. No other environmental exposure factors were recorded. According to the study's protocol children with symptoms of a respiratory tract infection and children who were immunized with one or more doses of any pneumococcal vaccine were excluded from the study.

### Sample collection

Nasopharyngeal (NP) swabs were collected using a flexible nasopharyngeal flocked swab (Copan, Italy no 503SC01) as recommended by WHO [14],[18]. Swabs were placed into 1.0 ml of skim milk tryptone glucose glycerol (STGG) transport medium, shipped on wet ice directly to the Eijkman Institute, Jakarta. Upon arrival at the lab, 20 µl of each STGG sample was plated onto a 5% sheep blood agar supplemented with 5 mg/L gentamicin (SB-Gent), and incubated at 35°C for 24 h with 5% CO_2_. In the case of alpha-hemolytic colonies growth on the SB-Gent plate, a single colony was re-cultured and tested by Gram-staining, and also tested for susceptibility to optochin [13]. Gram-positive, optochin-sensitive isolates were stored in STGG at −80°C for further analysis.

### DNA extraction

Bacterial DNA was extracted as described previously [19]. Briefly, pneumococcal isolates were retrieved from storage by subculture on the SB-Gent. The bacterial cells suspension was heated at 100°C for 10 minutes and instantly frozen at −20°C for 10 minutes. Lysates were centrifuged at 1000×g for 10 minutes, after which the supernatant was collected and stored at −20°C until further use.

### Molecular detection of pneumococcal surface antigen A gene

The polymerase chain reaction (PCR) targeting the pneumococcal surface antigen A gene (*psa*A) was performed as described by Morrison et al. [20]. In short, the reaction mixture contained GoTaq Green Master Mix (Promega), forward (5′-CTTTCTGCAATCATTCTTG-3′) and reverse (3′-GCCTTCTTTACCTTGTTCTGC-5′) primers at 10 µM concentration, and 1.0 µl of DNA template. The presence of 838 bp amplicon was detected by electrophoresis of 5 µl of PCR product on 1% agarose gels stained with ethidium bromide, and visualized in UV light.

### Serotyping

Serotype determination was performed by a sequential multiplex PCR (smPCR), as published by Pai *et al.* [19]. Briefly, seven smPCRs were performed, each in a 25 µl reaction mixture of GoTaq Green Master Mix (Promega) and up to five pairs of primers specific for a particular serotype or serotypes cluster and an internal positive control targeting 160 bp fragment of capsule transcriptional regulator gene *wzg* (*cps*A) universally present in *cps* operons of almost all serotypes and using 1.0 µl of cell lysate extract as DNA template. The primers set used in the study allowed for identification of 40 serotypes, including all serotypes targeted by PCV13 and were published by the CDC (USA) [21].

### Antibiotic susceptibility testing

Antibiotic susceptibility tests were carried out for all of the pneumococcus isolates using the disk diffusion method according to CLSI standard [22], and antimicrobial disks (Oxoid) with chloramphenicol, clindamycin, erythromycin, sulfamethoxazole/trimethoprim, and tetracycline. Susceptibility to penicillin was tested with the oxacillin disk [22]. Strains expressing lack of susceptibility to three or more antimicrobial agents of different classes were considered multidrug resistant (MDR) in the study.

### Statistical methods

Statistical analyses were conducted using GraphPad Prism V5.0 (GraphPad Software, San Diego, CA, USA).

## Results

*Streptococcus pneumoniae* isolates were cultured from 41 of 90 (46%) nasopharyngeal samples collected in the study from children infected with HIV in Jakarta, Indonesia. All strains were susceptible to optochin and positive for the *psa*A gene by PCR. The patient characteristics are described in Table 1. There were no differences in carriage rates within gender, family size, use of antibiotics, or tobacco smoking in the household. Although, *S. pneumoniae* carriage rates were higher in children with a CD4 lymphocyte count less than 25% (59%), compared to children with a CD4 count >25% (37%) the difference did not reach statistical significance (Fisher exact test *p* = 0.086) (Table 1). There was no correlation between child age and CD4 count (Pearson; *r* = 0.09, *p* = 0.46) neither of the differences in carriage rates between age groups in the study were significant. There were no exclusions from the study based on a child's previous immunization with any pneumococcal vaccine.

**Table 1:** Patient characteristics related to pneumococcal carriage.

Characteristics		N	N (%) of children carrying *S. pneumoniae*
**HIV-infected children**		90	41 (46)
**Age (month)**			
	0–24	9	3 (33)
	25–60	33	15 (46)
	61–144	48	23 (48)
**Sex**			
	Male	44	18 (49)
	Female	46	23 (51)
**Exposure to cigarette**			
	Yes	41	18 (44)
	No	49	23 (47)
**No of family member**			
	[1]–[3]	34	17 (50)
	[4]–[6]	28	11 (39)
	[>7]	12	6 (50)
	no data	16	7 (44)
**Current antibiotics use**			
	Yes	20	9 (45)
	No	70	32 (46)
**CD4 lymphocyte count** a			
	<25%	34	20 (59)
	≥ 25%	30	11 (37)
	no data	26	10 (39)

a CD4 lymphocyte count measured within 3 months prior to nasopharyngeal sampling.

Altogether, we cultured 42 *S. pneumoniae* strains from 41 samples, with a single sample from one child positive simultaneously for strains of serotype 3 and 9V. The most commonly observed was serotype 19F (8 of 42 cultured pneumococcal strains; 19%) followed by 9A and 6A/B (4 carriers each; 10%), 23F (3 carriers; 7%), 9V, 35B, 11A (two carriers each; 5%) and serotypes 18C, 3, 12F, 15B/C and 35F (single carrier each; 2%) (Table 2). We found that eleven isolates (26%) were untypeable using the SM-PCR method, with six of those 11 (14% of all) also being PCR-negative for the *cpsA* gene. In this study, strains that could be covered by the pneumococcal conjugate vaccine varied between 45% to 60% for PCV7 and PCV13 vaccines, respectively.

**Table 2:** Serotype distribution and vaccine coverage among 42 *S. pneumoniae* carriage isolates of HIV-infected children in Jakarta.

Serotype		N (%) of isolates
**19F**		8 (19)
**19A**		4 (10)
**6A/B**		4 (10)
**23F**		3 (7)
**11A**		2 (5)
**9V**		2 (5)
**sg18**		2 (5)
**12F**		1 (2)
**15B/C**		1 (2)
**3**		1 (2)
**35B**		1 (2)
**35F**		1 (2)
**7F**		1 (2)
**untypeable**		
	cps-positive	5 (12)
	cps-negative	6 (14)
**PCV-7 coverage**		19 (45)
**PCV-13-coverage**		25 (60)

The majority of strains were susceptible to chloramphenicol (86%), clindamycin (79%), erythromycin (76%), sulphamethoxazole/trimethoprim (41%) and tetracycline (43%) (Table 3). Meanwhile, only 33% of strains were susceptible to penicillin (Table 3). Use of the oxacillin disc to screen isolates for lack of susceptibility to penicillin could be considered as a limitation in our study as it does not allow to distinguish low level from high level resistance with low level resistant strains often retaining sensitivity to a range of beta-lactams, including aminopenicllins [22]. Compared to strains of other serotypes, isolates of PCV13 serotypes detected in the study (3, 6A/B, 7F, 9V, 14, 18C, 19A, 19F, and 23F) were less susceptible to any of the six antimicrobial agents tested, although the difference was significant only for penicillin (Table 3). In this study, we found 16 of isolates expressed a lack of susceptibility to three or more antimicrobial agents of different classes thus considered multi-drug resistant (MDR) (Table 4). With 13 (52%) of 25 strains of PCV13 serotypes versus three (18%) of 17 non-PCV13 serotype strains classified as MDR in our study, the multidrug resistance was more common, however the difference did not reach statistical significance (Fisher exact test *p* = 0.0504) among isolates of serotypes targeted by the vaccine.

**Table 3:** Antimicrobial susceptibility of *Streptococcus pneumoniae* strains carried by children infected with HIV.

Antimicrobial Agent	Number (%) of susceptible isolates			*p*-Value (Fisher exact test)
	All (n = 42)	PCV13 serotype strains^a^ (n = 25)	non-PCV13 serotype strains (n = 17)	
Chloramphenicol	36 (86)	20 (80)	16 (94)	0.3739
Clindamycin	33 (79)	18 (72)	15 (88)	0.2708
Erythromycin	32 (76)	16 (64)	16 (94)	0.0312
Sulphamethoxazole/trimethoprim	17 (41)	7 (28)	10 (59)	0.0605
Penicillin^b^	14 (33)	5 (20)	9 (53)	0.0448
Tetracycline	18 (43)	8 (32)	10 (59)	0.1169

a Strains of serotypes targeted by thirteen-valent conjugated polysaccharide pneumococcal vaccine: 1, 3, 4, 5, 6A, 6B, 7F, 9V, 14, 18C, 19A, 19F, 23F.

b Susceptibility to penicillin was determined with oxacillin disk [22].

**Table 4:** Serotype of multi-drug resistant *S. pneumoniae* strains.

Isolate	Serotype	Antimicrobial susceptibility profile [22]					
		Chloramphenicol	Clindamycin	Erythromycin	Sulphamethoxazole/trimethoprim	Penicillin^a^	Tetracycline
ISID-77	19F	S	R	R	R	R	R
ISID-107	19F	S	S	R	R	R	R
ISID-1	19F	S	R	R	R	R	R
ISID-16	19F	S	R	R	R	R	R
ISID-31	19F	S	S	R	R	R	R
ISID-12	19F	S	R	R	R	R	R
ISID-8	19A	S	S	S	R	R	R
ISID-6	19A	S	S	S	R	R	R
ISID-24	19A	S	S	S	R	R	R
ISID-110	6A/B	S	R	R	R	R	R
ISID-11	6A/B	S	R	R	R	R	R
ISID-36	12 F	R	S	S	R	S	R
ISID-47	11A	S	R	R	R	S	R
ISID-104	23F	R	R	R	R	R	R
ISID-75-R	9V	R	S	S	R	R	R
ISID-111	untypeable	S	R	S	R	S	R

S – susceptible; R – non-suceptible.

a Susceptibility to penicillin was determined with oxacillin disk [22].

## Discussion

Since limited data was available on the epidemiology of *S. pneumoniae* carriage in the Indonesian population, especially in high-risk children, we studied *S. pneumoniae* carriage in children infected with HIV. Our findings of 46% of *S. pneumoniae* carriage in HIV-positive children (aged 4 to 144 months) in Jakarta are in line with a previously published report on carriage in healthy children in Lombok Island and Semarang, Indonesia, where 48% of children (aged 0–25 months) and 43% of children (aged 6–60 months) carried pneumococi [15],[17]. *S. pneumoniae* carriage in children with this acquired immunodeficiency varies in different geographical locations. In comparison to other studies in which nasopharyngeal carriage of *S. pneumoniae* was detected in HIV-infected children using the WHO-recommended culture method, the prevalence of pneumococcal carriage in Jakarta was lower compared to 66% recorded in Tanzania (children aged 12–168 months) and 77% reported in Kenya (3–59 months) [23],[24], but higher than in Romania (children aged 39–106 months), Brazil (0–228 months), and USA with the reported carriage rates of 30%, 29%, and 20%, respectively [25]–[27]. There is relatively little known about possible impact of the HIV infection on pneumococcal carriage. Abdullahi *et al.* [24] reported higher carriage prevalence in Kenya among HIV-positive versus HIV-negative children whereas infection with HIV has no effect on pneumococcal carriage reported in adults in South Africa by Shiri *et al.* [28]. Although we observed a trend towards higher *S. pneumoniae* carriage rates in children with lower CD4 lymphocyte count, both Mwenya, *et al.* [29] and Anthony *et al.* [23] reported lack of any association between CD4 levels and pneumococcal carriage in HIV-infected children.

Pneumococcal conjugate vaccines are reported to provide substantial protection against IPD and clinical pneumonia when given to HIV-infected infants [30]. Despite PCV7 vaccine being available in Indonesia since 2008 and PCV13 since 2011, their use is limited as it is evident from lack of any exclusion from the study based on child previous immunization against pneumococcal disease but also from a relatively high prevalence of vaccine serotypes in carriage. We observed that serotype 19F isolates were the most common in carriage in this study. Meanwhile in 2001, Soewignjo *et al*. reported that in healthy children from Lombok, Indonesia, the most common were strains of serogroup 6 (25%) followed by serogroup 23 (21%) and serogroup 19 (6%) [15]. Farida *et al.* reported that in healthy children from Semarang, Indonesia, the most common were strains of serotype 6A/B (19%) followed by serotype 15B/C and 11A (10%), 23F(9%), and 19F(8%) [17]. In our study, serogroup 19 isolates (serotypes 19F and 19A together) accounted for over a quarter (12 out of 42 or 29%) of all the pneumococcal strains cultured from HIV-infected children. Interestingly, eleven isolates were classified as untypeable in the study, with six strains of PCR-negative for the *cpsA* gene. It either indicates over-representation of untypeable strains when carriage is detected by conventional culture [31], reflects significant circulation of strains expressing capsular types not targeted by SM-PCR, or indicates low sensitivity of the protocol used to determine the serotype of pneumococcal strains.

We identified susceptibility to sulfamethoxazole/trimethoprim in 41%, and to penicillin in 33% carriage isolates in this study, whereas susceptibility to sulfamethoxazole/trimethoprim and penicillin was still common (91% and 100% respectively) in the study conducted in 1997 in Lombok [15]. Meanwhile in Semarang, Indonesia in 2010, 24% of *S. pneumoniae* strains were penicillin non-susceptible, and 45% were resistant to sulfamethoxazole/trimethoprim [17] . We also found that serotype 19F isolates along isolates of serogroup 6A/B were more frequently resistant to antimicrobial drugs tested in the study compared to strains of other serotypes. This is in agreement with ANSORP (Asian Network for Surveillance of Resistant Pathogens) data reporting a 59% multidrug resistance among *S. pneumoniae* invasive isolates collected in the region, with 19F being the major multidrug resistant serotype (24% of all MDR strains from IPD) [32]. Furthermore, recent Malaysian data showed that serotype 19F was correlated with increased resistance against penicillin [33].

We observed strains of serotypes targeted by PCV13 to be more frequently resistant to antipneumococcal drugs tested in the study compared to non-PCV13 strains. Immunization with PCVs would target not only serotypes common in carriage in the studied population, but also strains of serotypes less susceptible to anti-pneumococcal drugs. In geographical locations with high rates of antibiotics resistance among *S. pneumoniae* strains, introduction of PCVs lowered not only incidence of IPD, but also lowered (at least temporarily) rates of resistance to particular antimicrobial agents in strains circulating in carriage and causing pneumococcal diseases [34]–[36]. Similar effects could be expected in our study population. In conclusion, our study gives insight into the population of *S. pneumoniae* strains circulating in carriage in patients who are at high risk for IPD due to age and comorbidity. We expect our results to be helpful in shaping preventive strategies targeting IPD in Indonesia both on a national and local level.
